# Nurses’ AI training acceptance and tool usage: a structural equation model of individual and hospital-level factors

**DOI:** 10.3389/fpubh.2026.1864635

**Published:** 2026-07-14

**Authors:** Linli Guo, Qin Ding, Mengjie Tang, Siyun Wei, Limei Chen, Xiaotao Cao, Xiaoxia Zhang, Zuli Zheng

**Affiliations:** 1 Department of Clinical Trial Center, West China Hospital, Sichuan University/West China School of Nursing, Sichuan University, Chengdu, Sichuan, China; 2 Department of Mental Health Center, National Center for Mental Disorders, West China Hospital, Sichuan University/West China School of Nursing, Sichuan University, Chengdu, Sichuan, China; 3 Department of Colorectal Cancer Center, West China Hospital, Sichuan University/West China School of Nursing, Sichuan University, Chengdu, Sichuan, China; 4 Key Laboratory of Birth Defects and Related Diseases of Women and Children, Ministry of Education, Department of Pediatrics Nursing, West China Second University Hospital, Sichuan University, Chengdu, Sichuan, China; 5 Department of Neurology, Qinghai Provincial People’s Hospital, Xining, China; 6 Department of Women and Child Psychiatry, The Second People’s Hospital of Guizhou Province, Guiyang, China

**Keywords:** anxiety, artificial intelligence, attitude, hospital, tool usage, training acceptance

## Abstract

**Objectives:**

With the rapid advancement of artificial intelligence (AI), it has exerted a profound influence on the medical field. Currently, AI applications in nursing remain nascent in China. This study aimed to investigate nurses’ attitudes and anxiety levels toward AI in general hospitals in western China, and to analyze the association of these psychological factors with their AI training acceptance and clinical AI tool usage behavior.

**Methods:**

A multicenter cross-sectional design was employed. In February 2026, a questionnaire survey was conducted among 620 registered nurses in public general hospitals across western China (Sichuan, Guizhou, and Qinghai provinces). The questionnaire collected data on nurses’ demographic information, whether they had received AI training and whether they had used AI tools in the workplace, as well as their responses to the General Attitudes Toward AI Scale (GAAIS) and the AI Anxiety Scale (AIAS). Participation was voluntary and anonymous. Descriptive statistics, reliability and validity testing, correlation analysis, and structural equation modeling were performed using R software (version 4.5.1).

**Results:**

Of 603 returned questionnaires (97.3% response rate), 591 were valid. Negative attitudes were significantly associated with lower training acceptance (*β* = −0.140, *p* = 0.015) and lower tool usage (*β* = −0.141, *p* = 0.003). Positive attitudes and AI anxiety showed no significant associations with either outcome. Older age and longer work experience were associated with higher rates of both outcomes, with work experience showing the strongest association with tool usage. No significant association was found between training acceptance and tool usage after covariate adjustment (*β* = 0.070, *p* = 0.356).

**Conclusion:**

Hospital-level resources, older age, and longer work experience were the factors most strongly associated with nurses’ AI training acceptance and AI tool usage, with work experience showing the strongest association with tool usage. Negative attitudes were associated with lower engagement in both outcomes, whereas positive attitudes and anxiety showed no significant independent associations. These findings suggest that institutional support, experience-based peer learning, and targeted reduction of negative perceptions may be key priorities for AI integration in nursing practice.

## 1 Introduction

Artificial intelligence (AI) is a collective term for technologies that enable computers to mimic human intelligent behaviors, including perception, reasoning, learning, and decision-making. AI encompasses key technologies such as machine learning, speech recognition, image recognition, natural language processing, and expert systems (1). Worldwide, AI is increasingly reshaping the healthcare sector, with applications ranging from disease prediction and diagnosis to medical imaging, drug monitoring, patient management, and clinical decision support (2). In nursing, AI applications include patient data monitoring and predictive analytics, AI-assisted diagnostic systems, automated patient monitoring devices, and clinical decision support for nurses (3–5). This enables early intervention for potential complications and adverse events, thereby enhancing patient safety, optimizing workflows, improving care quality, and supporting personalized patient care. Furthermore, AI can reduce nurses’ administrative burden by automating documentation tasks such as charting and record-keeping, freeing up time for direct patient care and potentially improving patient outcomes (6). Recognizing the dual potential and challenges of AI integration, leading international nursing organizations—including the International Council of Nurses (ICN), the American Nurses Association (ANA), and the American Organization for Nursing Leadership (AONL)—have collectively endorsed digital transformation in the nursing profession (7). They emphasize that this digital transformation could improve patient outcomes while alleviating workforce shortages and economic pressures on healthcare systems. Nurses, as the largest segment of the healthcare workforce, are at the forefront of these technological changes.

Recent years have witnessed a marked increase in AI research within the nursing field. Surveys of nursing students have revealed that positive attitudes toward AI correlate with an increased willingness to adopt the technology (8). Research by Tarsuslu et al. revealed that positive attitudes toward AI among nurses were significantly correlated with both higher readiness to adopt AI in nursing practice and lower concern about its application (9). Yıldırım et al. suggested that nurses’ AI-related understanding, willingness to learn, and confidence in using AI were critical determinants of their technological preparedness (10). Kaplan and Uçar surveyed nurses in three Turkish provinces and reported that nurses held generally positive attitudes toward AI, although significant regional differences existed. Most nurses believed that AI would replace humans, yet they were still willing to continue using AI tools (11). Despite the potential of AI in nursing, its implementation is hindered by numerous practical challenges. A Chinese study revealed that although most nurses demonstrated openness to AI applications, nearly all participants expressed concerns—primarily regarding reliability, implementation costs, and ethical considerations (12). Another study identified broader concerns among nurses about AI, extending beyond ethical dilemmas to include job replacement, workplace surveillance, data and patient safety, and the risk of technological misuse (13).

The attitudes, social influence, and perceived confidence and competence identified in the above research align closely with the core framework of the Theory of Planned Behavior (TPB). The TPB posits that behavioral intention—jointly determined by attitude, subjective norm, and perceived behavioral control—is the immediate antecedent of action. Perceived behavioral control reflects an individual’s perception of the ease or difficulty of performing the intended action. Within the TPB framework, emotion is primarily an antecedent variable that exerts an indirect effect on behavior through attitudes. However, when faced with emerging technologies such as AI that may carry occupational threats, anxiety may serve not merely as an attitudinal by-product but also as a factor independently associated with behavior. Uçar et al. reported that AI anxiety was positively associated with unemployment anxiety among university students (14). Traditionally, anxiety has been viewed as a negative emotion that can weaken self-efficacy, reduce perceived behavioral control, and consequently suppress behavioral intentions. Conversely, some psychologists contend that anxiety can motivate individuals to overcome their limitations, producing positive effects that foster behavioral intentions (15). Recent research suggests that enhanced AI training may be associated with reduced anxiety and more positive attitudes among nurses (16).

A growing body of research has investigated attitudes toward AI and AI-related literacy among nursing students, nurses, and other healthcare professionals (12, 17). However, few studies have simultaneously explored nurses’ attitudes toward AI and their anxiety. Therefore, grounded in the TPB framework, this study aimed to investigate the relationship between nurses’ attitudes toward AI and their anxiety levels in general hospitals in western China. Specifically, this study examined: (a) the associations between nurses’ cognitive attitudes (positive and negative) toward AI and their AI training acceptance and AI tool usage behavior; (b) the associations between AI-related anxiety and the two behavioral outcomes; (c) the role of demographic and hospital-level factors in these associations; and (d) the association pattern between AI training acceptance and AI tool usage behavior. This study contributes to the development of training programs and strategies designed to support AI integration and advancement in nursing practice.

## 2 Methods

### 2.1 Research design and study participants

This multicenter, cross-sectional study was conducted in three public general hospitals in western China in February 2026. The participating hospitals included West China Hospital of Sichuan University, Qinghai Provincial People’s Hospital, and Guizhou Provincial Second People’s Hospital. Ethical approval was obtained from the Biomedical Ethics Review Committee of West China Hospital of Sichuan University (Approval Number: 2024–1778). Inclusion criteria were: (a) voluntary participation, and (b) being a registered nurse in practice. Exclusion criteria were: (a) unwillingness to participate or withdrawal during the study, (b) incomplete questionnaire responses, and (c) age under 18 years. Prior to data collection, participants received detailed information about the purpose and significance of the study and subsequently provided written informed consent. Participation was voluntary, and participants were free to withdraw at any stage. Data were collected via convenience sampling, with participants completing paper-based questionnaires within a designated time frame. This study used a questionnaire comprising 51 items, consisting of sections on general information, the GAAIS, and the AIAS. The sample size was determined based on the rule of 5 to 10 times the number of variables, with an additional 20% allowance for invalid responses, resulting in a target enrollment of 306 to 612 participants. Of the 620 questionnaires distributed, 603 were returned (response rate: 97.26%), of which 591 were considered valid.

### 2.2 Data collection instrument

The study used the General Information Questionnaire for Nurses, the General Attitudes Toward AI Scale (GAAIS), and the AI Anxiety Scale (AIAS) as data collection instruments.

#### 2.2.1 General information for nurses

The questionnaire comprised two sections. The first section included 10 items: eight assessing socio-demographic characteristics (age, gender, educational level, marital status, years of experience, job title, hospital, and department) and two pertaining to nurses’ AI training and AI usage experience.

#### 2.2.2 General attitudes toward artificial intelligence scale

The GAAIS was developed by Astrid Schepman and Paul Rodway to measure people’s attitudes toward AI (18). The GAAIS consists of two subscales with a total of 20 items—12 assessing positive attitudes and 8 assessing negative attitudes. Responses were recorded on a five-point Likert scale, ranging from 1 (strongly oppose) to 5 (strongly agree). Negative items were reverse-scored, while positive items were coded as originally worded. Higher total scores indicate more favorable attitudes toward AI, and lower scores indicate less favorable attitudes. The Chinese version of the scale was translated by Qin Zeng (19). After validation, the Chinese version of the scale showed good internal consistency and structural validity. In this study, the Cronbach’s *α* coefficient was 0.889 for the positive dimension of the GAAIS and 0.799 for the negative dimension. The Kaiser-Meyer-Olkin (KMO) value was 0.913 for the positive dimension and 0.841 for the negative dimension.

#### 2.2.3 Artificial intelligence anxiety scale

The AIAS was developed by Professor Yu-Yin Wang to evaluate AI-related anxiety (20). The AIAS consists of 21 items across four dimensions: learning, job replacement, sociotechnical blindness, and AI configuration. The Cronbach’s α coefficients for these dimensions were 0.974, 0.917, 0.917, and 0.961, respectively. All coefficients exceeded the 0.7 threshold, indicating sound internal consistency. Items were rated on a 7-point Likert scale, yielding total scores ranging from 21 to 147. Higher scores correspond to higher levels of AI anxiety. In this study, the Cronbach’s α coefficients for the AIAS subscales—learning, job replacement, sociotechnical blindness, and AI configuration—were 0.931, 0.861, 0.873, and 0.876, respectively, with the total scale coefficient being 0.931. All subscale Cronbach’s α coefficients exceeded 0.7, meeting the fundamental psychometric requirement for internal consistency. Furthermore, the KMO values for the AIAS subscales ranged from 0.738 to 0.919, with an overall KMO of 0.924, all exceeding the 0.7 threshold.

### 2.3 Statistical analyses

This study employed descriptive statistical analysis. Categorical data on nurses’ demographic and occupational characteristics were described using frequencies (percentages), while continuous data—including dimension and total scores of the GAAIS and AIAS—were presented as means ± standard deviations. Group comparisons were performed based on participants’ receipt of AI training and use of AI tools. Pearson’s correlation analysis was used to examine associations between continuous variables, and Spearman’s rank correlation analysis was used for categorical variables, thereby clarifying the distributional characteristics and correlations among all study variables. First, reliability and validity tests were conducted on the GAAIS and AIAS. Internal consistency was assessed using Cronbach’s α and standardized Cronbach’s α coefficients. Construct validity was evaluated using the KMO test and Bartlett’s test of sphericity, and confirmatory factor analysis (CFA) was employed to assess the fit between the factor structure and the empirical data.

Based on these analyses, we fitted a structural equation model (SEM). The structural equation model was fitted using the Generalized Least Squares (GLS) estimator in the lavaan package of R, which is suitable for the current dataset containing continuous latent indicators, binary observed variables, and dummy categorical variables.

Missing data handling: Case-wise deletion (listwise deletion) was adopted. Participants with missing values on any included variable were directly excluded to retain a complete and authentic dataset; no multiple imputation or other missing data interpolation methods were used.

Modification indices: The SEM framework was constructed strictly based on theoretical hypotheses. Modification indices were not calculated or applied to adjust paths or error correlations, avoiding data-driven model revision.

Latent variable operationalization: Three latent variables were established:

AI anxiety (AIAS) was reflected by four dimensional total scores (learning anxiety, job replacement anxiety, sociotechnical blindness, and AI configuration anxiety);

Positive AI attitude was operationalized with 12 scale items;

Negative AI attitude was operationalized with 8 scale items.

Binary AI training and AI tool use behaviors, age, and working years were included as observed variables. Hospital category was converted into dummy variables (Taking medical institutions in Sichuan Province as the reference group) and incorporated into the model as confounding covariates.

A two-tailed test was used with a significance level of 0.05, and *p* < 0.05 were considered statistically significant. All statistical analyses were performed using R software (version 4.5.1).

## 3 Results

This study employed a cross-sectional survey design, with a sample of 591 registered nurses (including clinical nurses and nursing managers) recruited from public general hospitals in Sichuan, Guizhou, and Qinghai provinces, China. The sample included participants from Sichuan (*N* = 184), Guizhou (*N* = 229), and Qinghai (*N* = 178). It aimed to systematically investigate the mechanisms linking nurses’ perceptions and attitudes toward AI in clinical practice, their levels of AI-related anxiety, and their behaviors regarding AI training uptake and use of AI tools in the workplace.

### 3.1 Intergroup differences in baseline characteristics and Core variables

Table 1 presents the demographic and occupational characteristics and core scale scores of participants, stratified by AI training status and AI tool usage in the workplace. This identified variables with significant intergroup differences and provided empirical evidence for variable selection in the subsequent SEM analysis. Of the 591 total participants, 439 (74.28%) were in the non-training group and 152 (25.72%) in the training group; 292 (49.41%) were in the non-use group and 299 (50.59%) in the use group.

**Table 1:** Description of the basic characteristics and each dimension scores of the artificial intelligence questionnaire for the interviewed nurses.

Variables	Overall (*N* = 591, 100.00%)	Whether received any AI training			Whether ever used AI tools		
		No (*N* = 439, 74.28%)	Yes (*N* = 152, 25.92%)	*p-*values	No (*N* = 292, 49.41%)	Yes (*N* = 299, 50.59%)	*P-*values
Age							
18–30 years	259 (43.824)	198 (45.103)	61 (40.132)	0.091	137 (46.918)	122 (40.803)	0.295
31–40 years	273 (46.193)	204 (46.469)	69 (45.395)		129 (44.178)	144 (48.161)	
> 40 years	59 (9.983)	37 (8.428)	22 (14.474)		26 (8.904)	33 (11.037)	
Sex							
Female	534 (90.355)	396 (90.205)	138 (90.789)	0.959	259 (88.699)	275 (91.973)	0.227
Male	57 (9.645)	43 (9.795)	14 (9.211)		33 (11.301)	24 (8.027)	
Educational level							
Junior college and below	47 (7.953)	37 (8.428)	10 (6.579)	0.581	28 (9.589)	19 (6.355)	0.193
Bachelor’s degree or above	544 (92.047)	402 (91.572)	142 (93.421)		264 (90.411)	280 (93.645)	
Marital status							
Unmarried	189 (31.980)	146 (33.257)	43 (28.289)	0.303	94 (32.192)	95 (31.773)	0.983
Married or divorced	402 (68.020)	293 (66.743)	109 (71.711)		198 (67.808)	204 (68.227)	
Work years							
< 3 years	85 (14.382)	60 (13.667)	25 (16.447)	0.211	38 (13.014)	47 (15.719)	0.025*
3–5 years	102 (17.259)	80 (18.223)	22 (14.474)		64 (21.918)	38 (12.709)	
6–9 years	123 (20.812)	98 (22.323)	25 (16.447)		61 (20.890)	62 (20.736)	
≥ 10 years	281 (47.547)	201 (45.786)	80 (52.632)		129 (44.178)	152 (50.836)	
Work position							
Primary nurse	492 (83.249)	371 (84.510)	121 (79.605)	0.204	244 (83.562)	248 (82.943)	0.927
Head nurse or manager	99 (16.751)	68 (15.490)	31 (20.395)		48 (16.438)	51 (17.057)	
Hospital							
Hospital A	184 (31.134)	58 (13.212)	126 (82.895)	<0.001*	0 (0.000)	184 (61.538)	<0.001*
Hospital B	229 (38.748)	203 (46.241)	26 (17.105)		114 (39.041)	115 (38.462)	
Hospital C	178 (30.118)	178 (40.547)	0 (0.000)		178 (60.959)	0 (0.000)	
GAAIS scale							
Positive dimension	3.475 ± 0.574	3.473 ± 0.527	3.480 ± 0.695	0.891	3.481 ± 0.549	3.469 ± 0.598	0.789
Negative dimension	2.459 ± 0.569	2.619 ± 0.492	1.997 ± 0.523	<0.001*	2.784 ± 0.457	2.141 ± 0.483	<0.001*
AIAS scale							
Learning	25.481 ± 8.394	27.938 ± 7.155	18.382 ± 7.650	<0.001*	31.164 ± 6.029	19.930 ± 6.437	<0.001*
Job replacement	24.281 ± 6.537	24.640 ± 6.355	23.243 ± 6.952	0.023*	25.726 ± 6.268	22.870 ± 6.495	<0.001*
AI configuration	10.171 ± 3.726	10.704 ± 3.582	8.632 ± 3.718	<0.001*	11.455 ± 3.487	8.916 ± 3.524	<0.001*
Social technology blind spot	16.756 ± 4.642	16.886 ± 4.544	16.382 ± 4.910	0.248	17.514 ± 4.389	16.017 ± 4.769	<0.001*
Total score	76.689 ± 18.389	80.169 ± 17.413	66.638 ± 17.462	<0.001*	85.860 ± 16.079	67.732 ± 15.943	<0.001*

AI, Artificial intelligence; GAAIS, The general attitudes toward artificial intelligence scale; AIAS, The artificial intelligence anxiety scale; *indicates a *P* < 0.05. Hospital A = Sichuan; Hospital B = Guizhou; Hospital C = Qinghai.

#### 3.1.1 Comparative analysis of sociodemographic characteristics across groups

No statistically significant differences were observed in gender, educational level, or marital status between the AI training groups or between the AI tool usage groups (all *p* > 0.05). These results suggest that the examined demographic characteristics were not key correlates of nurses’ AI training acceptance or AI tool usage; accordingly, they were not included as confounding factors in the subsequent SEM analysis. The age difference between the AI training and non-training groups was marginally significant (*p* < 0.1), suggesting a potential association; therefore, age was retained for subsequent analyses.

#### 3.1.2 Intergroup differences in occupational characteristics

A statistically significant difference in years of work experience was observed between the AI tool usage group and the non-usage group (*p* = 0.025). The proportion of nurses with 3–5 years of experience was significantly higher in the non-usage group (21.918%) than in the usage group (12.709%), whereas the proportion with ≥10 years of experience was significantly higher in the usage group (50.836%) than in the non-usage group (44.178%). Although no significant intergroup difference was observed for AI training (*p* = 0.211), this factor was ultimately included as a confounding control variable in the SEM due to its significant association with one of the core outcome variables.

No statistically significant differences were observed between nurses and nursing managers in terms of job position (*p* > 0.05), and this variable was therefore not included in subsequent modeling. Across different hospitals, significant differences were found in both AI training acceptance and AI tool usage (*p* < 0.001). The training acceptance rate was 82.895% in Sichuan Province, where AI tool utilization reached 100%; in Guizhou Province, the training acceptance rate was 17.105% and AI tool usage was 38.462%; notably, no nurses in Qinghai Province had received AI training or engaged in any AI tool usage. Hospital-level factors—namely, AI resource allocation, training infrastructure, and clinical application development—emerged as the primary correlates of variation in nurses’ behaviors. These variables were therefore accounted for as confounding factors within the SEM framework.

#### 3.1.3 Comparative analysis of core scale scores across groups

##### 3.1.3.1 General attitudes toward artificial intelligence scale

No significant group differences were observed in positive dimension scores (*p* = 0.891 for training groups, *p* = 0.789 for usage groups; both > 0.05). In contrast, negative dimension scores showed significant group differences (*p* < 0.001), with the non-training and non-usage groups yielding substantially higher scores (2.619 ± 0.492 vs. 1.997 ± 0.523; 2.784 ± 0.457 vs. 2.141 ± 0.483). These findings suggest a significant negative association between nurses’ negative perceptions of AI and both their engagement in AI training and their use of AI tools.

##### 3.1.3.2 Artificial intelligence anxiety scale

With the exception of the sociotechnical blindness dimension (*p* = 0.248 > 0.05), significant differences were found in the learning anxiety, job replacement anxiety, and AI configuration anxiety dimensions, as well as in the total score (all *p* < 0.05), with the non-training group scoring significantly higher. For the AI tool usage groups, all dimensions of the AIAS and the total score showed significant differences (*p* < 0.001), with the non-usage group scoring significantly higher. These findings suggest that nurses’ AI-related anxiety, particularly concerning learning and job replacement, is significantly correlated with both their AI training acceptance and their use of AI tools.

In summary, based on the analysis in this section, the GAAIS and AIAS were designated as core explanatory variables, and age, years of experience, and hospital affiliation as confounding variables for the subsequent SEM analysis, which was then used to construct a multi-path SEM.

#### 3.1.4 Psychometric evaluation results for the scale

As shown in Tables 2, 3, reliability, construct validity, and CFA support the suitability of the two scales for nursing populations.

**Table 2:** The reliability and validity test results of each dimension of the GAAIS and AIAS scales.

Variables	Cronbach alpha	Std cronbach alpha	KMO test	Bartlett’s test of sphericity statistical value	Bartlett’s test of sphericity *p*-value
GAAIS					
GAAIS positive	0.889	0.892	0.913	2910.093	< 0.001*
GAAIS negative	0.799	0.805	0.841	1389.644	< 0.001*
AIAS					
Learning	0.931	0.933	0.919	4259.146	<0.001*
Job replacement	0.861	0.862	0.817	1652.592	<0.001*
Social technology blind spot	0.873	0.873	0.830	1166.025	<0.001*
AI configuration	0.876	0.876	0.738	918.401	<0.001*
Total score	0.931	0.931	0.924	9402.786	<0.001*

AI, Artificial intelligence; GAAIS, The general attitudes toward artificial intelligence scale; AIAS, The artificial intelligence anxiety scale; KMO test, Kaiser-Meyer-Olkin test; *indicates a *P* < 0.001.

**Table 3:** The confirmatory factor analysis results of the GAAIS and AIAS scales.

Variables	Chi-square test value	df	Chi-square test df	RMSEA	CFI	TLI	SRMR
GAAIS positive dimension	391.277	54	7.246	0.103	0.883	0.857	0.055
GAAIS negative dimension	169.355	20	8.468	0.112	0.891	0.848	0.068
AIAS	1282.071	183	7.006	0.101	0.882	0.865	0.078

GAAIS, The general attitudes toward artificial intelligence scale; AIAS, The artificial intelligence anxiety scale; RMSEA, The root mean square error of approximation; CFI, Comparative fit index; TLI, Tucker-Lewis index; SRMR, Standardized Root Mean Square Residual.

##### 3.1.4.1 Reliability analysis

This study used Cronbach’s *α* and standardized Cronbach’s α to assess the internal consistency of the questionnaire. For the GAAIS, α = 0.889 for the positive dimension and α = 0.799 for the negative dimension. For the AIAS, the α coefficients for the four dimensions were: learning anxiety (α = 0.931), job replacement anxiety (α = 0.861), sociotechnical blindness (α = 0.873), and AI configuration anxiety (α = 0.876); the overall scale reliability was α = 0.931. All dimension α coefficients exceeded 0.7, meeting the psychometric threshold for internal consistency. The Cronbach’s alpha coefficients for the learning anxiety dimension and the total scale were both > 0.9, indicating excellent reliability, good internal consistency among items within each dimension, and stable and dependable measurement results.

##### 3.1.4.2 Assessment of construct validity

Construct validity was examined using the Kaiser-Meyer-Olkin (KMO) test and Bartlett’s test of sphericity. The KMO values for the positive and negative dimensions of the GAAIS were 0.913 and 0.841, respectively. For the AIAS, KMO values for the four dimensions ranged from 0.738 to 0.919, with an overall scale value of 0.924. All KMO values exceeded the 0.7 threshold, and Bartlett’s test of sphericity was significant (*p* < 0.001), indicating sufficient common factors for factor analysis. These findings demonstrate adequate structural validity and satisfy the prerequisites for subsequent CFA and SEM analyses.

##### 3.1.4.3 Confirmatory factor analysis

This section evaluates the alignment between the scale’s theoretical factor structure and the study data. For the positive dimension of the GAAIS, χ^2^/df = 7.246, RMSEA = 0.103, SRMR = 0.055; for the negative dimension, χ^2^/df = 8.468, RMSEA = 0.112, SRMR = 0.068. For the AIAS, χ^2^/df = 7.006, RMSEA = 0.101, SRMR = 0.078. All SRMR values were below the recommended cutoff of 0.08. All χ^2^/df values were below the commonly used threshold of 10 for large-scale cross-sectional studies. For the GAAIS, the positive dimension yielded CFI = 0.883 and TLI = 0.857, while the negative dimension yielded CFI = 0.891 and TLI = 0.848. The AIAS demonstrated CFI = 0.882 and TLI = 0.865. However, the RMSEA values for all scales exceeded the conventional cutoff of ≤0.08, indicating suboptimal absolute fit. Given that CFI/TLI approached or slightly exceeded 0.85 and SRMR and χ^2^/df were within acceptable ranges, the measurement model was considered adequate for subsequent path analysis. The dimensional structure of the scales showed partial support in the nursing population and can be used for latent variable construction in subsequent SEM.

#### 3.1.5 Results of inter-variable correlation analysis

**Figure 1:**
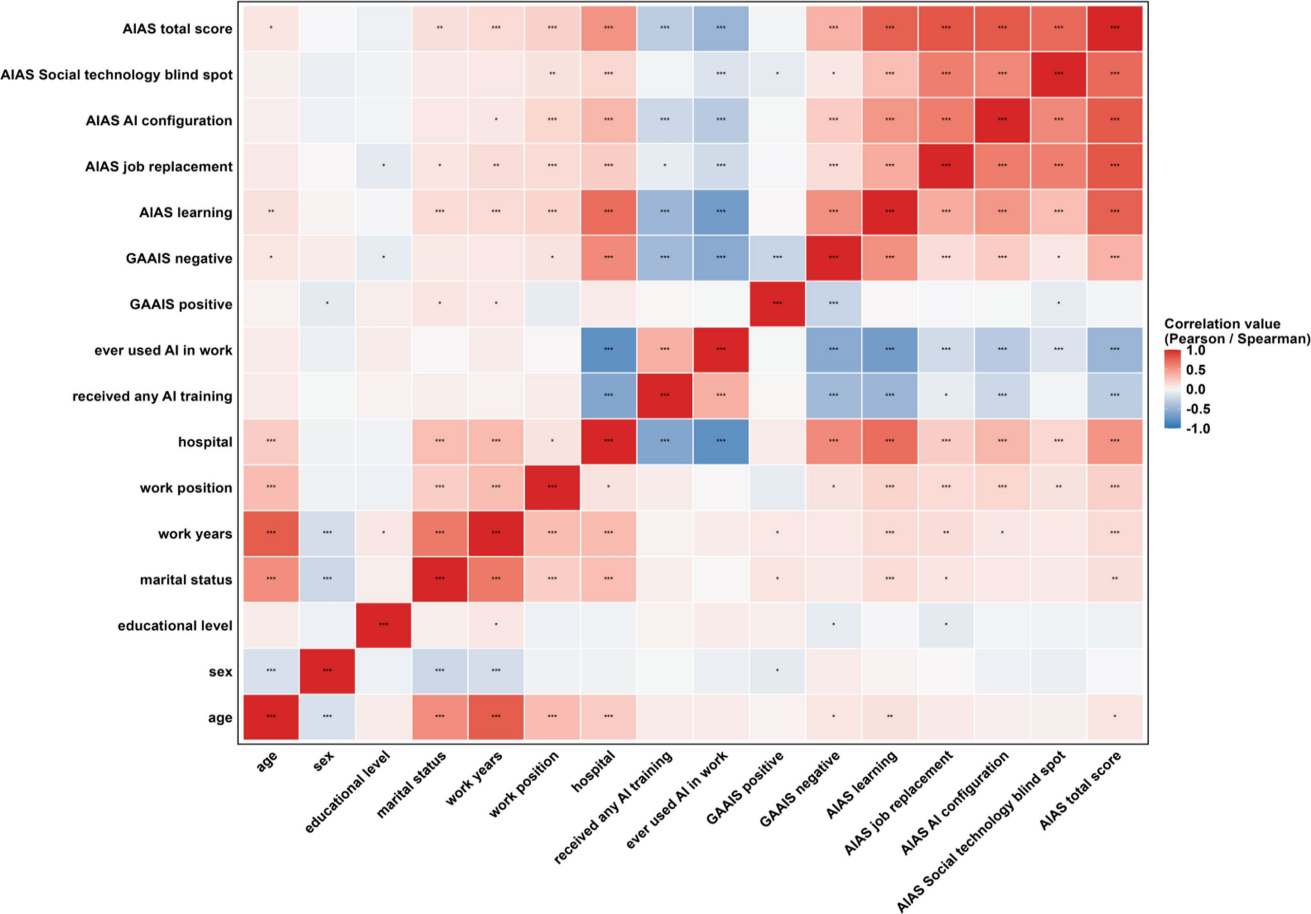
Correlation matrix of different attitudes toward artificial intelligence and AI-related anxiety dimensions among hospital nursing staff. Categorical variables were analyzed using Spearman’s rank correlation coefficient, whereas continuous variables were using Pearson’s product- moment correlation coefficient. ***p < 0.001, **p < 0.01, *p < 0.05.

Pearson’s product–moment correlation coefficients were calculated for continuous variables, and Spearman’s rank correlation coefficients for categorical variables, to generate a full correlation matrix for all study variables. As shown in Figure 1, the core outcome variables—acceptance of AI training and use of AI tools—had significant negative associations with the negative dimension of the GAAIS (*p* < 0.05), with each dimension of the AIAS (*p* < 0.01), and with the total AIAS score (*p* < 0.001), whereas no significant correlation was observed with the positive dimension of the GAAIS. This is fully consistent with the intergroup comparison results in Table 1, further confirming the direction of associations among the core variables.

Among the confounding factors, age, years of work experience, and hospital affiliation were significantly correlated with both core outcome variables (*p* < 0.05), whereas gender, educational level, marital status, and job position showed no statistically significant correlations with the outcome variables, providing further empirical support for the inclusion of confounding factors in the subsequent SEM. Furthermore, regarding the dimensions of the scales, the positive and negative dimensions of the GAAIS showed a significant negative correlation, while all dimensions of the AIAS exhibited significant positive correlations with one another. These patterns are consistent with the theoretical construction logic of the scales, confirming the validity of the relationships among the variables and providing a correlational basis for the path hypotheses in the SEM.

#### 3.1.6 Structural equation model

##### 3.1.6.1 Overall model fit

To address the issues of estimation method applicability and parameter bias arising from extreme covariates in the initial model, the robust maximum likelihood (MLR) estimator was employed in place of the generalized least squares (GLS) estimator to better accommodate the non-normal distribution of the binary outcome variables. In addition, hospital dummy variables with extremely stratified distributions were removed to reduce potential distortion of the core associations, yielding a final model that focused on the association patterns between individual-level psychological characteristics and AI training acceptance and AI tool usage behavior Table 4.

**Table 4:** The path coefficients of structural equation model for the interrelationships among AI training, AI usage, different attitudes toward AI, and the AI anxiety scale.

Path of the variables	Coefficients	Standard error	*Z-*values	Standard coefficients	95% CI of Standard coefficients		*P*-values
					Lower	Upper	
Bidirectional influence path							
AIAS and GAAIS positive dimension	−0.029	0.051	−0.561	−0.029	−0.129	0.072	0.575
AIAS and GAAIS negative dimension	−0.063	0.054	−1.184	−0.063	−0.168	0.042	0.236
GAAIS positive and negative dimension	−0.432	0.044	−9.928	−0.432	−0.518	−0.347	<0.001
Received any AI training and ever used AI tools	0.010	0.011	0.924	0.070	−0.011	0.031	0.356
Unidirectional influence path							
GAAIS positive dimension to received any AI training	−0.009	0.017	−0.551	−0.028	−0.042	0.024	0.581
GAAIS negative dimension to received any AI training	−0.046	0.019	−2.421	−0.140	−0.083	−0.009	0.015
AIAS to received any AI training	0.022	0.015	1.460	0.068	−0.008	0.052	0.144
GAAIS positive dimension to ever used AI tools	−0.006	0.018	−0.332	−0.022	−0.041	0.029	0.740
GAAIS negative dimension to ever used AI tools	−0.039	0.013	−2.985	−0.141	−0.064	−0.013	0.003
AIAS to ever used AI tools	−0.006	0.012	−0.514	−0.022	−0.029	0.017	0.607
Confounding factors influence path							
Age to received any AI training	0.122	0.033	3.734	0.218	0.058	0.185	<0.001
Work years to received any AI training	0.050	0.020	2.493	0.148	0.011	0.089	0.013
Age to ever used AI tools	0.102	0.025	4.040	0.218	0.052	0.151	<0.001
Work years to ever used AI tools	0.118	0.015	7.644	0.418	0.088	0.148	<0.001

As shown in Table 5, the revised model yielded the following fit indices: χ^2^ = 2511.299, df = 339, χ^2^/df = 7.408; RMSEA = 0.104 (95% CI: 0.100–0.108); GFI = 0.764, CFI = 0.700, TLI = 0.666, SRMR = 0.095. Given that hospital-level variables, which were strongly associated with between-group variance in the outcomes, were removed from the model, the decline in overall fit indices was expected. All core path directions and significance levels remained consistent with the descriptive analyses and theoretical expectations.

**Table 5:** Fit indices of the structural equation model.

Chi-square test value	Chi-square test df	RMSEA	95% CI of RMSEA		GFI	CFI	TLI	SRMR
			Lower	Upper				
2511.299	339	0.104	0.100	0.108	0.764	0.700	0.666	0.095

##### 3.1.6.2 Residual covariance paths among variables

**Figure 2:**
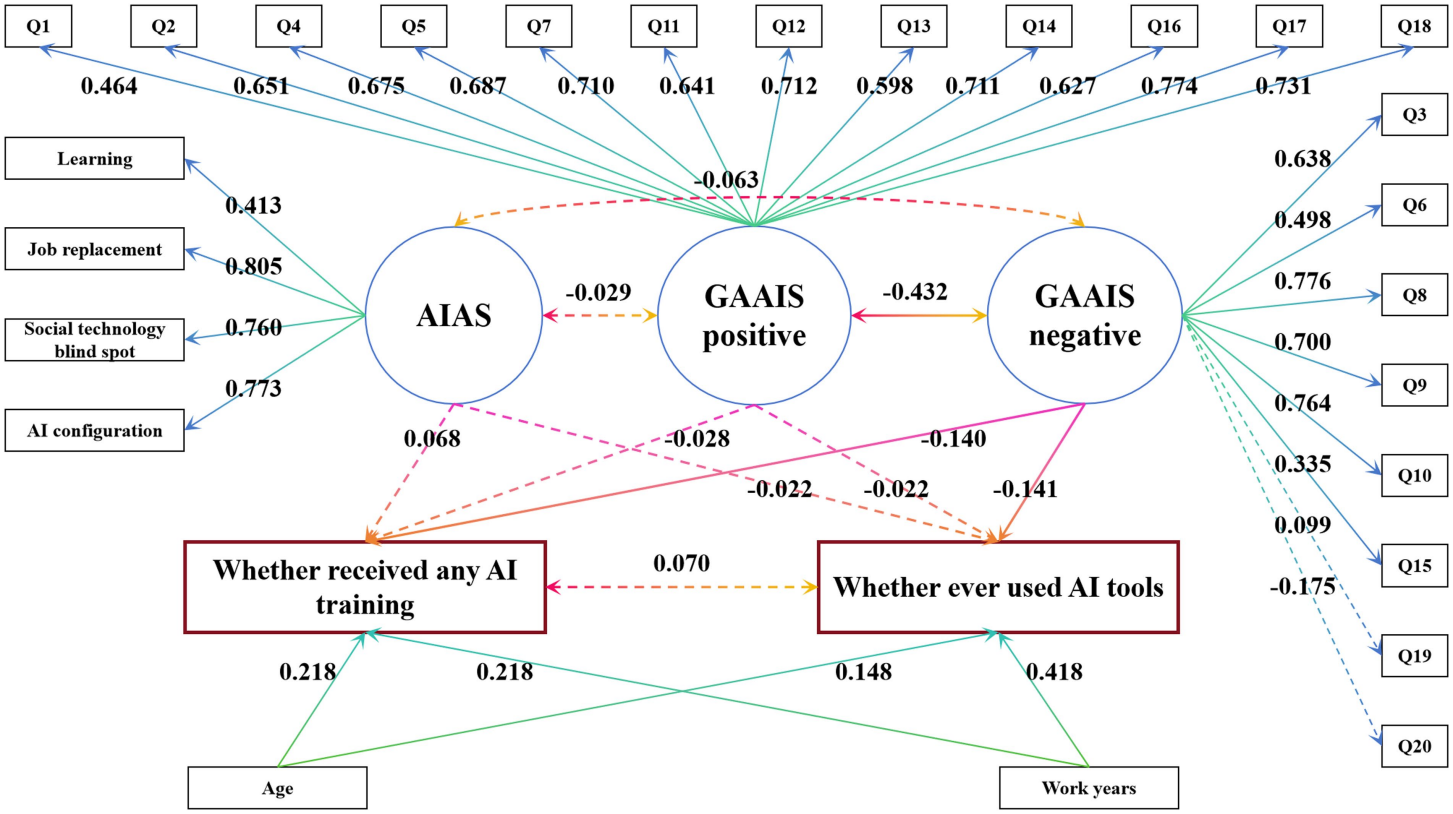
The structural equation model path diagram for the interrelationships among AI training, AI usage, different attitudes towards AI, and the AI anxiety scale.

As illustrated in Figure 2, the SEM path diagram provides a visual summary of the direct associations and residual covariances among the key variables.

###### 3.1.6.2.1 Residual covariances among latent variables

The positive and negative dimensions of the GAAIS showed a highly significant negative residual association (*β* = −0.432, *p* < 0.001), indicating an inverse relationship between the two: higher levels of positive cognitive attitudes toward AI were associated with lower levels of negative cognitive attitudes. This pattern aligns with the theoretical construction logic of the scale.

The residual associations between the AIAS total score and the GAAIS positive dimension (*β* = −0.029, *p* = 0.575) and negative dimension (*β* = −0.063, *p* = 0.236) were both statistically non-significant, indicating that, after controlling for demographic variables, AI-related anxiety showed no independent associations with cognitive attitudes toward AI. The overall correlations between the two were largely explained by shared demographic factors, suggesting that they may be treated as relatively independent psychological constructs.

###### 3.1.6.2.2 Residual covariance between outcome variables

The residual covariance between AI training acceptance and AI tool usage behavior was positive but not statistically significant (*β* = 0.070, *p* = 0.356), suggesting that the positive association between the two at the bivariate level was primarily explained by shared factors—including negative cognitive attitudes, age, years of work experience, and hospital-level resource allocation. After accounting for these factors, no additional independent association was observed between the two behavioral variables.

##### 3.1.6.3 Direct associations of core explanatory variables

This section examines the direct associations of AI-related cognitive attitudes and anxiety levels with the two outcome variables. All results are consistent with the group comparison findings presented in Table 1.

###### 3.1.6.3.1 Direct associations with AI training acceptance

The GAAIS negative dimension showed a significant negative direct association (*β* = −0.140, *p* = 0.015), indicating that higher levels of negative cognitive attitudes were associated with a lower probability of accepting AI training.

However, neither the GAAIS positive dimension (*β* = −0.028, *p* = 0.581) nor the AIAS total score (*β* = 0.068, *p* = 0.144) showed significant direct associations with training acceptance, suggesting that negative attitudes—rather than positive attitudes or anxiety—are the primary psychological correlate of training acceptance.

###### 3.1.6.3.2 Direct associations with AI tool usage behavior

**S**imilarly, the GAAIS negative dimension showed a highly significant negative direct association with AI tool usage behavior (*β* = −0.141, *p* = 0.003), indicating that higher levels of negative cognitive attitudes were associated with a lower probability of using AI tools in clinical practice.

In parallel, neither the GAAIS positive dimension (*β* = −0.022, *p* = 0.740) nor the AIAS total score (*β* = −0.022, *p* = 0.607) showed significant direct associations with AI tool usage behavior. These results reveal a consistent pattern across the two outcome variables: negative cognitive attitudes showed a significant direct association, whereas positive attitudes and anxiety levels did not exhibit independent direct associations. This pattern suggests that, among the psychological constructs examined, negative perceptions were the primary correlate of AI-related behavioral outcomes.

##### 3.1.6.4 Direct associations of control variables

The direct associations of age and years of work experience with the two outcome variables were as follows.

Age showed highly significant positive direct associations with both AI training acceptance (*β* = 0.218, *p* < 0.001) and AI tool usage behavior (*β* = 0.218, *p* < 0.001), indicating that older nurses had a higher probability of both accepting AI training and using AI tools.

Years of work experience also showed significant positive direct associations with AI training acceptance (*β* = 0.148, *p* = 0.013) and AI tool usage behavior (*β* = 0.418, *p* < 0.001). Notably, the effect size for work experience on AI tool usage behavior was the largest among all variables (*β* = 0.418), suggesting a relatively strong positive association between clinical experience and AI tool usage behavior.

## 4 Discussion

Based on cross-sectional survey data from 591 nurses in three general hospitals in western China, this study examined the associations between nurses’ cognitive attitudes toward AI, AI-related anxiety, and their AI training acceptance and clinical AI tool usage behavior. The findings revealed that negative cognitive attitudes toward AI were negatively associated with nurses’ AI training acceptance and clinical AI tool usage behavior. In contrast, positive cognitive attitudes and AI-related anxiety did not exhibit independent associations with either outcome after covariate adjustment. Additionally, older age and longer work experience were positively associated with both behavioral outcomes. Furthermore, between-hospital disparities in AI resource allocation emerged as organizational-level factors that were strongly associated with the differentiation of the two behavioral outcomes.

The above findings suggest that nurses’ AI training acceptance and tool usage behavior are associated with multiple factors rather than a single determinant. The relationship between training acceptance and tool usage does not reflect a simple direct association, but appears to be linked through shared antecedent factors. Furthermore, psychological factors—attitudes and anxiety—showed differentiated patterns of association with the outcomes. At the organizational and individual levels, hospital resources and clinical experience emerged as more fundamental external correlates.

### 4.1 Association between AI training acceptance and AI tool usage behavior

At the descriptive level, AI training acceptance and AI tool usage behavior showed a significant positive correlation at the bivariate level; however, after controlling for shared factors—including negative cognitive attitudes, age, years of work experience, and hospital-level resource allocation—their residual covariance did not reach statistical significance. This pattern indicates that the positive correlation between training acceptance and tool usage at the bivariate level is primarily attributable to shared factors, rather than reflecting a direct independent association between the two. These findings suggest that improving both training acceptance and tool usage may require supportive conditions at both the organizational and individual levels, rather than single-component interventions. Karaarslan et al. similarly reported that structured training significantly improved nurses’ AI knowledge but had limited effects on attitudes and behavioral change, suggesting that training does not automatically translate into behavioral change (21). This aligns with our findings, which together suggest that organizational and individual conditions may be necessary for training effects to materialize.

### 4.2 General attitudes toward AI and behavioral outcomes

This study found that the positive and negative dimensions of the GAAIS showed differentiated association patterns with nurses’ AI training acceptance and AI tool usage behavior. Negative cognitive attitudes were negatively associated with both outcomes, indicating that higher levels of negative attitudes toward AI were associated with lower likelihood of training participation and tool usage. In contrast, positive cognitive attitudes showed no significant associations with either outcome. Berşe et al. reported a weak positive correlation between the positive and negative subscales of the GAAIS, indicating that positive and negative cognitive attitudes toward AI are not opposite ends of a continuum but rather independent psychological constructs that may coexist (22). Hanci and Gok Metin similarly reported that the negative dimension of the GAAIS was significantly negatively correlated with several psychological variables, while the positive dimension showed no significant associations with occupational stress or related measures (23). The aforementioned studies all support the view that positive and negative attitudes have a differential association pattern with individual psychological and behavioral indicators, which is consistent with the findings of this study.

Regarding negative cognitive attitudes, our SEM results showed that negative attitudes were significantly and negatively associated with both nurses’ AI training acceptance and AI tool usage behavior. Specifically, nurses who had not received AI training scored significantly higher on negative attitudes than those who had received training, and non-users of AI tools also scored significantly higher than users. This result is consistent with previous literature. Amin et al. found that there is a positive correlation between negative attitudes and resistance behaviors (24). Akca Sumengen et al. reported that the increase in AI awareness and usage is associated with a decrease in negative attitudes (25). Şimşek et al. reported that negative attitudes are one of the major factors influencing nursing students’ clinical AI competence (26). Yu et al. surveyed Chinese nurses and found that, in resource-limited primary healthcare settings, negative dimension scores were substantially higher than positive dimension scores (27). Taken together, these studies indicate a consistent association pattern between negative attitudes and reduced AI-related behavioral engagement.

In contrast to the significant associations observed for negative attitudes, positive attitudes showed no independent associations with nurses’ AI training acceptance or AI tool usage behavior in this study. This is consistent with Sengul et al., who found that positive attitudes alone did not independently promote self-directed learning (28). Similarly, Busch et al. and Sarhan S, et al. reported that high positive attitudes coexisted with low actual usage among medical students in multinational and Egyptian samples, respectively (29, 30). Together, these studies suggest that positive attitudes alone may not be sufficient to be associated with behavioral engagement. Based on a multicenter sample from western China, this study similarly showed that AI resources were unevenly distributed across hospitals, and that positive attitudes were not accompanied by higher behavioral engagement when nurses lacked training opportunities or institutional support. El Arab et al. emphasized the important role of organizational support and other factors in AI adoption, suggesting that the influence of positive attitudes may be context-dependent (31). The present study similarly found that positive attitudes were less strongly associated with behavioral engagement when nurses perceived insufficient training opportunities or organizational support.

The above findings can be understood from the perspective of the TPB. According to the theory, attitude is one of the key correlates of behavioral intention. Although the cross-sectional design of this study does not allow for testing the full pathway described by the TPB, the observed association between negative attitudes and lower behavioral engagement aligns with the theoretical predictions of the TPB, suggesting that negative cognitions may be an important psychological factor associated with reduced AI-related behavioral engagement among nurses. Intervention strategies could prioritize reducing nurses’ negative cognitions rather than focusing solely on enhancing their positive attitudes toward AI.

### 4.3 Associations of AI-related anxiety with training acceptance and tool usage

Another psychological factor closely associated with attitude is AI anxiety. In both group comparisons and correlation analyses, AI-related anxiety was significantly negatively associated with nurses’ AI training acceptance and AI tool usage behavior, indicating that higher levels of AI anxiety were linked to lower behavioral engagement. However, when the model accounted for negative cognitive attitudes and demographic characteristics (age, years of work experience), the independent association of AI anxiety ceased to be significant. This suggests that the bivariate association between AI anxiety and behavior may largely reflect its covariation with negative attitudes or shared demographic factors.

The significant associations between AI anxiety and behavior observed in group comparisons and correlation analyses are consistent with previous literature. Ayed et al. found that anxiety levels were significantly higher among non-users than among AI users, which aligns with the direction of the group differences observed in our study (32). However, our SEM results further revealed that the independent association of anxiety became non-significant after negative cognitive attitudes were incorporated into the model. This finding is consistent with Kwak et al., who reported that AI anxiety indirectly influenced behavioral intention through attitudes and self-efficacy (33). Kanat et al. further found that negative GAAIS attitude scores were moderately to strongly positively correlated with all AIAS dimensions, suggesting a close covariation between attitudes and anxiety (34). Similarly, Liu et al. found that AI anxiety was significantly negatively correlated with the total GAAIS score, and that anxiety played a partial mediating role between AI literacy and attitudes (16). Collectively, these studies suggest that anxiety and attitudes do not function independently as psychological factors; when both are entered simultaneously into a model, the associations of anxiety may be overridden by those of attitudes. Taken together with our findings, this suggests that among nurses, AI anxiety and negative attitudes are closely intertwined, and their associations with behavior may be more indirectly expressed through attitudes rather than serving as direct independent correlates.

These findings suggest that when formulating AI clinical promotion strategies, managers may prioritize reducing nurses’ negative cognitions rather than focusing solely on addressing their anxiety. The alleviation of anxiety may be a natural consequence of attitude improvement rather than a direct driving force for behavioral change.

### 4.4 Associations of hospital-level factors with training acceptance and tool usage

Among the common factors associated with training and usage behaviors, disparities in resource allocation at the hospital level emerged as prominent organizational correlates. Descriptive analyses in this study revealed significant between-hospital differences in nurses’ AI training acceptance and AI tool usage behavior. In a medical institution in Sichuan Province (with 4,300 beds), the acceptance rate of AI training among nurses reached 82.9%, and the utilization rate of AI tools reached 100%. In a medical institution in Guizhou Province (with 1,200 beds), these two indicators were 17.1 and 38.5%, respectively. In a medical institution in Qinghai Province (with 2,200 beds), both of these two indicators were 0%. All three hospitals are Class III Grade A teaching hospitals, yet they showed stark disparities in AI infrastructure, training system development, and clinical application scenarios, highlighting the current uneven distribution of AI resources across hospitals in western China. Notably, the Qinghai hospital, despite having 2,200 beds (more than the 1,200-bed Guizhou hospital), showed a lower AI adoption rate. This suggests that hospital size alone does not determine AI resource allocation.

This structural disparity is consistent with previous research. Hwang et al. found that medical AI implementation in the U. S. was uneven, with higher adoption rates in resource-rich large medical centers than in resource-limited community hospitals (35). Gulamali et al. found that resource disparities were significantly correlated with differences in the application of AI among hospitals (36). Zeng et al. also found marked disparities in AI resource allocation across hospitals in Beijing, Sichuan, and Yunnan among Chinese nurses (19). These studies suggest that hospital-level resource accessibility is an organizational factor closely associated with nurses’ AI training and usage behaviors.

Hospital resources are closely associated with nurses’ behavioral choices and may also be indirectly linked through cognitive attitudes. Ramadan et al. pointed out that 70% of nurses cited inadequate infrastructure as the primary external barrier affecting their willingness to use AI (37). Buchanan et al. emphasized the significance of adequate resources and support systems for the effective utilization of artificial intelligence in clinical settings (38). Ronquillo et al. emphasized the crucial role of infrastructure and support in enabling the application of artificial intelligence in nursing (39). In the present study, nurses at the Qinghai hospital had access to neither AI training nor AI tools, and their behavioral engagement was closely associated with organizational resource availability rather than being solely determined by individual willingness or cognition. In resource-constrained settings, even when individuals hold positive attitudes or low negative cognitions, behavioral engagement remains closely linked to external conditions.

In summary, hospital-level AI resources were closely associated with nurses’ adoption of AI. Reducing between-hospital disparities may facilitate subsequent individual-level interventions.

### 4.5 Associations of age and work experience with AI training acceptance and tool usage behavior

Beyond organizational-level resource disparities, individual-level differences also emerged as important correlates of training and usage behaviors. Our SEM results showed that both age and years of work experience were consistently and positively associated with AI training acceptance and AI tool usage behavior, with work experience demonstrating the strongest association with tool usage. Descriptive data also showed that nurses with more extensive clinical experience had higher participation rates in AI training and higher usage rates of AI tools. Among AI tool users, nurses with ≥10 years of work experience accounted for 50.8%, significantly higher than the 44.2% in the non-user group. In contrast, nurses with 3–5 years of experience comprised only 12.7% of the user group, compared to 21.9% in the non-user group.

These findings are consistent with Qaladi et al., who reported that nurses with extensive clinical experience had higher usage rates of AI-assisted management tools than their less experienced colleagues (40). Abuzaid et al. also found that older nurses and nursing students with more years of experience showed a higher level of recognition regarding the positive correlation between AI and nursing practice (41). This association may reflect that more experienced nurses often manage complex tasks and clinical decisions, and may have greater functional needs for AI-assisted tools. Additionally, extensive clinical experience may support critical evaluation of AI’s strengths and limitations, which may in turn be related to higher AI adoption rates. Zhou et al. also found that junior nurses often lack systematic technical training, operational guidance, and emotional support mechanisms (42). In contexts where hospitals provide inadequate support, novice nurses may perceive AI as a technology imposed on them, and this perception is associated with lower willingness to adopt AI.

These findings suggest that healthcare institutions could establish peer learning mechanisms whereby senior nurses share their AI tool experiences in complex case decision-making, while junior nurses apply their digital literacy to support operational implementation, facilitating the complementary integration of experience and technology.

### 4.6 Implications for nursing practice

Hospital-level resource allocation was strongly associated with AI training and adoption among nurses, and its association was even stronger than individual psychological differences. Nurses’ negative attitudes toward AI were significantly associated with lower training acceptance, whereas positive attitudes and AI-related anxiety showed no significant independent associations with either outcome. Moreover, no significant association was observed between AI training acceptance and AI tool usage behavior.

This indicates that advancing the application of AI in nursing requires a concerted effort across three levels: institutional resources, training, and individual nurses. Policymakers can prioritize allocating resources to hospitals in underserved areas to narrow regional gaps, thereby providing a foundational guarantee for AI implementation. Hospitals can integrate AI training into nurses’ routine continuing education, providing them with opportunities to learn AI-related knowledge. AI training should be targeted, leveraging the experiential advantages of senior nurses by pairing them with younger nurses who have higher digital literacy, thereby promoting peer learning and achieving a complementary integration of experience and technology. Key components of AI training promotion should include dispelling nurses’ negative perceptions of AI, appropriately acknowledging their AI-related anxieties, and channeling these into motivation for learning.

### 4.7 Limitations

This study has several limitations. First, the sample was restricted to general hospitals in Sichuan, Guizhou, and Qinghai provinces and employed convenience sampling, which may limit the generalizability of the findings. Although the target population comprised nurses from various departments in general hospitals across western China, the non-random selection process may introduce selection bias, potentially affecting the objectivity of the conclusions. Second, this study relies on self-report questionnaires at a single time point to measure attitudes toward AI and AI-related anxiety. This may introduce common method bias, and the obtained results may overestimate the true correlations. Future research should adopt multi-source or longitudinal methods to alleviate this limitation. Third, this study is cross-sectional in nature, collecting data at a single time point. Therefore, it can only reflect the current status and correlations among the variables at that specific moment, without establishing causal relationships or temporal sequences. Another limitation is the significant differences among hospitals in training acceptance and AI tool usage rates. Although all three hospitals are tertiary teaching hospitals, they differ in bed capacity and geographic location, which may reflect unmeasured differences in resources and policies. Furthermore, some of the reported RMSEA values are relatively high. This may be partially attributable to the multi-center design, cultural differences across provinces, and the complexity of the constructed model. Future research could consider revising the scale items or exploring a factor structure that is more suitable for the nursing population (especially for multi-center heterogeneous samples) to further improve the model fit. Despite these limitations, this study employed valid and reliable measurement tools to explore nurses’ attitudes toward AI and their levels of AI-related anxiety in western China, thereby laying the groundwork for AI development in the nursing field. Future research should broaden the participant pool and conduct cross-regional studies to enhance generalizability. For more hospital-based studies, it is necessary to collect and formally examine the associations of these institutional factors, and conduct the analysis using a multilevel model. In addition, longitudinal studies are needed to clarify temporal and causal relationships among the variables.

## 5 Conclusion

This study shows that nurses’ acceptance of AI training and their use of AI tools are associated with multi-level factors, including organizational resources, individual psychological factors, and training-related mechanisms. In light of the current resource disparities, efforts to support the integration of AI into nursing care may benefit from focusing on bridging the gaps between institutions, while relating nurses’ anxieties to learning engagement through carefully designed training programs, and associating their clinical experience with the AI adoption.

## Data Availability

The raw data supporting the conclusions of this article will be made available by the authors, without undue reservation.

## References

[1] Cho KA Seo YH. Dual mediating effects of anxiety to use and acceptance attitude of artificial intelligence technology on the relationship between nursing students’ perception of and intention to use them: a descriptive study. BMC Nurs. (2024) 23:212. doi: 10.1186/s12912-024-01887-z, 38539198 PMC10976840

[2] Kwak Y Seo YH Ahn J-W. Nursing students’ intent to use AI-based healthcare technology: path analysis using the unified theory of acceptance and use of technology. Nurse Educ Today. (2022) 119:105541. doi: 10.1016/j.nedt.2022.105541, 36116387

[3] Hassan EA El-Ashry AM. Leading with AI in critical care nursing: challenges, opportunities, and the human factor. BMC Nurs. (2024) 23:752. doi: 10.1186/s12912-024-02363-4, 39402609 PMC11475860

[4] Nashwan AJ Gharib S Alhadidi M El-Ashry AM Alamgir A Al-Hassan M . Harnessing artificial intelligence: strategies for mental health nurses in optimizing psychiatric patient care. Issues Ment Health Nurs. (2023) 44:1020–34. doi: 10.1080/01612840.2023.2263579, 37850937

[5] Nashwan AJ Cabrega JA Othman MI Khedr MA Osman YM El-Ashry AM . The evolving role of nursing informatics in the era of artificial intelligence. Int Nurs Rev. (2025) 72:e13084. doi: 10.1111/inr.13084, 39794874 PMC11723855

[6] Almagharbeh WT Alfanash HA Alnawafleh KA Alasmari AA Alsaraireh FA Dreidi MM . Application of artificial intelligence in nursing practice: a qualitative study of Jordanian nurses’ perspectives. BMC Nurs. (2025) 24:92. doi: 10.1186/s12912-024-02658-6, 39863852 PMC11762109

[7] Phillips N Ives Erickson J O’Sullivan H Ahmed S. The digital future of nursing: implications for practice. Nurs Adm Q. (2025) 49:319–25. doi: 10.1097/NAQ.0000000000000694, 40876053

[8] Labrague LJ Aguilar-Rosales R Yboa BC Sabio JB de Los Santos JA. Student nurses’ attitudes, perceived utilization, and intention to adopt artificial intelligence (AI) technology in nursing practice: a cross-sectional study. Nurse Educ Pract. (2023) 73:103815. doi: 10.1016/j.nepr.2023.103815, 37922736

[9] Tarsuslu S Agaoglu FO Bas M. Can digital leadership transform AI anxiety and attitude in nurses? J Nurs Scholarsh. (2025) 57:28–38. doi: 10.1111/jnu.13008, 39086074 PMC11771702

[10] Yıldırım TÖ Karaman M. Development and psychometric evaluation of the artificial intelligence attitude scale for nurses. BMC Nurs. (2025) 24:441. doi: 10.1186/s12912-025-03098-6, 40264200 PMC12013020

[11] Kaplan M Uçar M. Attitudes of nurses toward artificial intelligence: a multicenter comparison. Work. (2025) 80:1380–6. doi: 10.1177/10519815241291668, 40297872

[12] Wang X Fei F Wei J Huang M Xiang F Tu J . Knowledge and attitudes toward artificial intelligence in nursing among various categories of professionals in China: a cross-sectional study. Front Public Health. (2024) 12:1433252. doi: 10.3389/fpubh.2024.1433252, 39015390 PMC11250283

[13] Bodur G Cakir H Turan S Seren AKH Goktas P. Artificial intelligence in nursing practice: a qualitative study of nurses’ perspectives on opportunities, challenges, and ethical implications. BMC Nurs. (2025) 24:1263. doi: 10.1186/s12912-025-03775-6, 41088159 PMC12522738

[14] Uçar M Çapuk H Yiğit MF. The relationship between artificial intelligence anxiety and unemployment anxiety among university students. Work. (2025) 80:701–10. doi: 10.1177/10519815241290648, 40172842

[15] Strack J Esteves F. Exams? Why worry? Interpreting anxiety as facilitative and stress appraisals. Anxiety Stress Coping. (2015) 28:205–14. doi: 10.1080/10615806.2014.931942, 24902852

[16] Liu S Xiao Y Nie M Yuan X Wang L Wang M . Nurses’ attitudes toward artificial intelligence: AI literacy as a predictor and the mediating effect of AI anxiety. BMC Nurs. (2025) 24:1511. doi: 10.1186/s12912-025-04142-1, 41272773 PMC12752257

[17] Özçevik Subaşi D Akça Sümengen A Semerci R Şimşek E Çakır GN Temizsoy E. Paediatric nurses’ perspectives on artificial intelligence applications: a cross-sectional study of concerns, literacy levels and attitudes. J Adv Nurs. (2025) 81:1353–63. doi: 10.1111/jan.16335, 39003632

[18] Schepman A Rodway P. Initial validation of the general attitudes towards artificial intelligence scale. Comput Hum Behav Rep. (2020) 1:100014. doi: 10.1016/j.chbr.2020.100014, 34235291 PMC7231759

[19] Zeng Q Huang X Zhu J Su S Hu Y Zhang X. Mechanisms of nurses’ AI use intention formation in Sichuan, Yunnan, and Beijing, China: mediating effects of AI literacy via self-efficacy-to-attitude pathways. Front Public Health. (2025) 13:1622802. doi: 10.3389/fpubh.2025.1622802, 40709030 PMC12287104

[20] Wang Y-Y Wang Y-S. Development and validation of an artificial intelligence anxiety scale: an initial application in predicting motivated learning behavior. Interact Learn Environ. (2022) 30:619–34. doi: 10.1080/10494820.2019.1674887

[21] Karaarslan D Kahraman A Ergin E. How does training given to pediatric nurses about artificial intelligence and robot nurses affect their opinions and attitude levels? A quasi-experimental study. J Pediatr Nurs. (2024) 77:e211–7. doi: 10.1016/j.pedn.2024.04.031, 38658302

[22] Berşe S Akça K Dirgar E Ağar A. Nursing students’ early attitudes towards the use of artificial intelligence and ChatGPT: an exploratory study. Int J Nurs Pract. (2025) 31:e70083. doi: 10.1111/ijn.70083, 41311300

[23] Hanci N Gok Metin Z. A structural equation modeling based on the conservation of resources theory: analyzing the relations between artificial intelligence attitude, employability, and career stress in nursing students. Nurse Educ Today. (2026) 162:107078. doi: 10.1016/j.nedt.2026.107078, 41855758

[24] Amin SM El-Gazar HE Zoromba MA El-Sayed MM Atta MHR. Sentiment of nurses towards artificial intelligence and resistance to change in healthcare Organisations: a mixed-method study. J Adv Nurs. (2025) 81:2087–98. doi: 10.1111/jan.16435, 39235193

[25] Akca Sumengen A Ozcevik Subasi D Cakir GN. Nursing students’ attitudes and literacy toward artificial intelligence: a cross-sectional study. Teach Learn Nurs. (2025) 20:e250–7. doi: 10.1016/j.teln.2024.10.022

[26] Şimşek E Kudubeş AA Semerci Şahin R. The predictive effect of nursing students’ attitudes and acceptance towards artificial intelligence on their clinical competencies. Teach Learn Nurs. (2025) 20:e806–14. doi: 10.1016/j.teln.2025.02.036

[27] Yu M Yu R Zhou M Fan X Geng R Ji J . A cross-sectional analysis of AI readiness and attitudes among nurses in resource-limited Chinese county hospitals. Front Digit Health. (2026) 8:1778627. doi: 10.3389/fdgth.2026.1778627, 41867482 PMC12999563

[28] Sengul T Sarikose S Uncu B Kaya N. The effect of artificial intelligence literacy on self-directed learning skills: the mediating role of attitude towards artificial intelligence: a study on nursing and midwifery students. Nurse Educ Pract. (2025) 88:104516. doi: 10.1016/j.nepr.2025.104516, 40857841

[29] Busch F Hoffmann L Truhn D Ortiz-Prado E Makowski MR Bressem KK . Global cross-sectional student survey on AI in medical, dental, and veterinary education and practice at 192 faculties. BMC Med Educ. (2024) 24:1066. doi: 10.1186/s12909-024-06035-4, 39342231 PMC11439199

[30] Sarhan S Badran A Ghalwash D Gamal Almalahy H Abou-Bakr A. Perception, usage, and concerns of artificial intelligence applications among postgraduate dental students: cross-sectional study. BMC Med Educ. (2025) 25:856. doi: 10.1186/s12909-025-07544-6, 40551097 PMC12186392

[31] El Arab RA Alshakihs AH Alabdulwahab SH Almubarak YS Alkhalifah SS Abdrbo A . Artificial intelligence in nursing: a systematic review of attitudes, literacy, readiness, and adoption intentions among nursing students and practicing nurses. Front Digit Health. (2025) 7:1666005. doi: 10.3389/fdgth.2025.1666005, 41079691 PMC12507812

[32] Ayed A Ejheisheh MA Al-Amer R Aqtam I Ali AM Othman EH . Insights into the relationship between anxiety and attitudes toward artificial intelligence among nursing students. BMC Nurs. (2025) 24:812. doi: 10.1186/s12912-025-03490-2, 40597186 PMC12211391

[33] Kwak Y Ahn J-W Seo YH. Influence of AI ethics awareness, attitude, anxiety, and self-efficacy on nursing students’ behavioral intentions. BMC Nurs. (2022) 21:267. doi: 10.1186/s12912-022-01048-0, 36180902 PMC9526272

[34] Kanat C Çetinkaya ZS Durak A. Future nurses’ attitudes and anxiety toward artificial intelligence: a cross-sectional study. Nurse Educ Today. (2026) 162:107033. doi: 10.1016/j.nedt.2026.107033, 41719711

[35] Hwang Y-M Ng MY Pillai M Sahai MP Hernandez-Boussard T. The landscape of AI implementation in US hospitals. Nature Health. (2026) 1:99–112. doi: 10.1038/s44360-025-00016-7

[36] Gulamali F Kim JY Pejavara K Thomas C Mathur V Eigen Z . Eliminating the AI digital divide by building local capacity. PLOS Digit Health. (2025) 4:e0001026. doi: 10.1371/journal.pdig.0001026, 41129488 PMC12548875

[37] Ramadan OME Alruwaili MM Alruwaili AN Elsehrawy MG Alanazi S. Facilitators and barriers to AI adoption in nursing practice: a qualitative study of registered nurses’ perspectives. BMC Nurs. (2024) 23:891. doi: 10.1186/s12912-024-02571-y, 39695581 PMC11654280

[38] Buchanan C Howitt ML Wilson R Booth RG Risling T Bamford M. Predicted influences of artificial intelligence on the domains of nursing: scoping review. JMIR Nurs. (2020) 3:e23939. doi: 10.2196/23939, 34406963 PMC8373374

[39] Ronquillo CE Peltonen L-M Pruinelli L Chu CH Bakken S Beduschi A . Artificial intelligence in nursing: priorities and opportunities from an international invitational think-tank of the nursing and artificial intelligence leadership collaborative. J Adv Nurs. (2021) 77:3707–17. doi: 10.1111/jan.14855, 34003504 PMC7612744

[40] Qaladi O Alshammari M Abdulrahim Almalki A. Artificial intelligence (AI) in nursing administration: challenges and opportunities. PLoS One. (2025) 20:e0319588. doi: 10.1371/journal.pone.0319588, 40168297 PMC11960905

[41] Abuzaid MM Elshami W Fadden SM. Integration of artificial intelligence into nursing practice. Health Technol. (2022) 12:1109–15. doi: 10.1007/s12553-022-00697-0, 36117522 PMC9470236

[42] Zhou Q Yang L Tang Y Yang J Zhou W Guan W . The mediation of trust on artificial intelligence anxiety and continuous adoption of artificial intelligence technology among primacy nurses: a cross-sectional study. BMC Nurs. (2025) 24:724. doi: 10.1186/s12912-025-03406-0, 40597194 PMC12211254

